# The quaternary structure of *Thermus thermophilus* aldehyde dehydrogenase is stabilized by an evolutionary distinct C-terminal arm extension

**DOI:** 10.1038/s41598-018-31724-8

**Published:** 2018-09-06

**Authors:** Kevin Hayes, Mohamed Noor, Ahmed Djeghader, Patricia Armshaw, Tony Pembroke, Syed Tofail, Tewfik Soulimane

**Affiliations:** 1 Department of Chemical Sciences, University of Limerick, Limerick, V94 T9PX Ireland; 2 Bernal Institute, University of Limerick, Limerick, V94 T9PX Ireland; 3 Physics Department, University of Limerick, Limerick, V94 T9PX Ireland

## Abstract

Aldehyde dehydrogenases (ALDH) form a superfamily of dimeric or tetrameric enzymes that catalyze the oxidation of a broad range of aldehydes into their corresponding carboxylic acids with the concomitant reduction of the cofactor NAD(P) into NAD(P)H. Despite their varied polypeptide chain length and oligomerisation states, ALDHs possess a conserved architecture of three domains: the catalytic domain, NAD(P)^+^ binding domain, and the oligomerization domain. Here, we describe the structure and function of the ALDH from *Thermus thermophilus* (ALDH_Tt_) which exhibits non-canonical features of both dimeric and tetrameric ALDH and a previously uncharacterized C-terminal arm extension forming novel interactions with the N-terminus in the quaternary structure. This unusual tail also interacts closely with the substrate entry tunnel in each monomer providing further mechanistic detail for the recent discovery of tail-mediated activity regulation in ALDH. However, due to the novel distal extension of the tail of ALDH_Tt_ and stabilizing termini-interactions, the current model of tail-mediated substrate access is not apparent in ALDH_Tt_. The discovery of such a long tail in a deeply and early branching phylum such as *Deinococcus-Thermus* indicates that ALDH_Tt_ may be an ancestral or primordial metabolic model of study. This structure provides invaluable evidence of how metabolic regulation has evolved and provides a link to early enzyme regulatory adaptations.

## Introduction

Found in both prokaryotes and eukaryotes, aldehyde dehydrogenases (ALDH) (EC 1.2.1.3) constitute a large family of NAD(P)-dependent enzymes with molecular weights of 50–60 kDa. The human genome encodes for 19 known ALDHs^1^, with the basic catalysis being the oxidation of various aldehydes to their corresponding carboxylic acids *via* the formation of a covalently bonded thiohemiacetal intermediate with the substrate^2,3^. The primary role of ALDH in humans and other mammals is the protection of the body from toxic compounds. For example, ALDH from liver mitochondria metabolizes acetaldehyde (ethanal), the oxidation product of ethanol by alcohol dehydrogenase, to acetic acid. In half of the Asian population, a single amino acid residue change inactivates the liver mitochondrial ALDH, causing severe alcohol intolerance. The drug disulfiram (Antabuse), which is used in the treatment of alcoholism, inhibits the liver mitochondrial ALDH^3^. In addition to its detoxification role, ALDH is also important for the biosynthesis of retinoic acid^4^ and GABA neurotransmitter metabolism^5^. In recent times ALDH have also become a prime target in cancer research due to the abnormal activity of human ALDH in cancer disease models^6^. Interestingly, this group of enzymes has a role in non-enzymatic conditions, such as osmotic stress reduction^7^ and mammalian cornea UV exposure protection^8^.

Enzymatically, mammalian ALDHs can broadly be classified as cytosolic (Class 1), mitochondrial (Class 2) and those expressed in tumor, stomach and cornea (Class 3)^6,9^. Nonetheless, the range of possible substrates and their corresponding affinity in prokaryotic enzymes preclude a similar classification.

Despite the availability of various ALDH superfamily structures (>140 released and >20 unreleased structures), much of the attention has been on mesophilic enzymes including those of human and the archetypal *Escherichia coli*^10^. This is primarily due to their respective multiple roles with non-redundant independent regulation. Interestingly, unlike many other systems, ALDH studies often originated in human enzymes rather than bacterial ones, leading to rather inaccurate descriptions of ‘atypical’ features. For example, the metabolically-specific lactaldehyde and phenylacetalaldehyde dehydrogenases from *E*. *coli* were only characterized robustly after the human ALDH1/2/3, and were found to have differing cofactor specificities, rate-limiting step and salt bridge tendencies^10^. Considering that there are ~ 300 different domain architectures within the ALDH-like clan (Pfam: CL0099), the substrate and cofactor specificity are far from easily predicted computationally. This is further complicated by different oligomerization states.

Through site-directed mutagenesis experiments, it is known that the catalysis occurs as a five-step reaction mediated by three highly-conserved residues Cys302, Lys192 and Glu268 (human ALDH2 numbering), which corresponds to Cys295, Lys182 and Glu261 in ALDH_Tt_. Glu268 acts as the general base necessary for Cys302 activation through deprotonation in both the dehydrogenase and esterase reactions^11^. This reaction consists of (i) activation of the catalytic Cys302 by a water-mediated proton abstraction by Glu268, (ii) a nucleophilic attack by the Cys302 thiolate group on the electrophilic aldehyde, (iii) formation of the tetrahedral thiohemiacetal intermediate (this deacylation step is the rate-limiting step in ALDH2, but not in others – *cf*.^12,13^) with concomitant hydride transfer to the NAD(P)^+^ pyridine ring, (iv) hydrolysis of the thioester intermediate from the previous step and finally (v) the dissociation of the reduced cofactor [NAD(P)H] and subsequent enzyme regeneration by NAD(P)^+^ binding. Steps (i) and (iv) very likely require a Glu268-bound water molecule to facilitate Cys302 deprotonation and the subsequent hydrolysis of the thioester intermediate^2^.

Recent studies on interesting ALDHs from (hyper) thermophilic bacteria and archaea, including *Geobacillus thermodenitrificans*^14^, *Pyrobaculum* sp. (PDB ID: 4H73 and 4NMJ) (no primary citation available), *Geobacillus thermoglucosidasius* (PDB ID: 5J78)^15^ and the robustly characterized glyceraldehyde-3-phosphate dehydrogenases from *Trichomonas tenax*^16^ and *Sulfolobus solfataricus*^17^, and the presence of native ALDH_Tt_ as a contaminant during *T*. *thermophilus caa*_3_*-*cytochrome oxidase crystallization^18^, motivated us to investigate ALDH_Tt_ biochemically and structurally.

In this article, we report the crystal structure of native ALDH_Tt_ and truncated mutants thereof. In contrast to other thermophilic ALDHs, but in agreement with mesophilic enzymes, the enzymatic characterization suggests the ability of ALDH_Tt_ to utilize both NAD^+^ and NADP^+^ as a cofactor. It also exhibits a broad range of substrate specificity. ALDH_Tt_ contains an unusually extended C-terminal arm forming novel interactions with the N-terminus in the quaternary structure which interacts closely with the substrate entry tunnel. The presence of a full complement of both N- and C-terminal residues in ALDH_Tt,_ in a deeply branching bacterial lineage such as *Thermus*, gives a structural snapshot of the early evolution of ALDH. This lends detail as to why terminal truncations were favored in evolutionarily more advanced ALDHs.

## Results

### ALDH_Tt_ is a tetrameric aldehyde dehydrogenase with unique structural features

**Figure 1:**
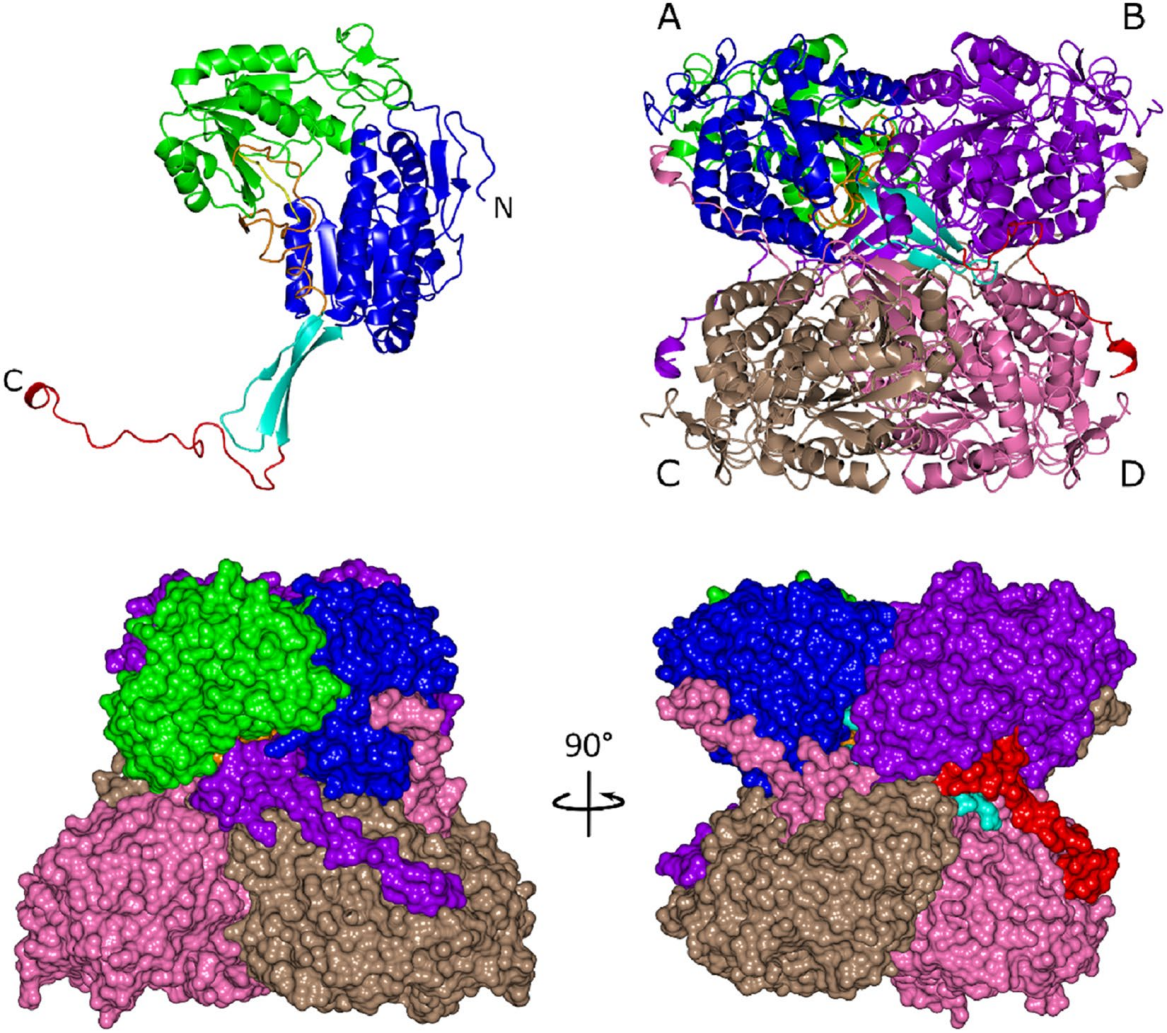
Top. ALDH_Tt_ monomeric and tetrameric domain architecture in ribbon illustration. Monomer A consists of the catalytic domain (green), NAD(P)^+^ binding domain (blue), short linker loop (yellow), inter-domain linker (orange), oligomerisation domain (cyan) and the C-terminal tail (red). Bottom. Surface representation of the tetrameric assembly of ALDH_Tt_ showing intimate relationship between protomers. Coloring for monomer A’s domains is kept consistent to aid orientation. Monomers B, C and D are colored in purple, light brown, and pink respectively.

The biologically active form of ALDH is a homotetramer. In the case of ALDH_Tt_, two copies related by a non-crystallographic symmetry (NCS) were present in the asymmetric unit. Based on PISA analysis^19^, however, it was clear that the tetramer assembly in ALDH_Tt_ consists of a combination of both NCS and crystallographic symmetry perpendicular to each other, forming a 222 symmetry (Fig. 1). In comparison, the calculated change in free energy values for the truncated mutants indicates a roughly linear trend towards destabilization of the tetrameric assembly. Nonetheless, this was not reflected in the analytical gel filtration elution (Supplementary Fig. S1). An interesting aspect of the PISA calculation is that both dimers and tetramers of ALDH_Tt_ are stable in solution (ΔG^diss^ > 30 kcal/mol), although the higher ΔG^int^ for tetrameric assembly shifts the thermal equilibrium away from a dimeric assembly.

PISA interface analysis shows strong interactions between the A-B dimer, with a buried surface area (BSA) of 3,500 Å^2^ out of a total of 22,900 Å^2^, created by a network of 32 hydrogen bonds and 13 salt bridges. The A-C interface exhibits a slightly weaker interaction with a BSA of 2,770 Å^2^ (8 hydrogen bonds and 12 salt bridges).

The most striking feature of the tetramer is the orientation of the C-terminal arm extension in the overall quaternary structure. It wraps around the outside of the symmetry related dimer pair (A + D or B + C in Fig. 1) creating a network of hydrogen bonds and salt bridges with the opposing monomers N-terminal residues and oligomerisation domain. This network of bonding pulls the tail across the substrate access tunnel opening i.e., the tail of monomer A covers the tunnel of monomer B.

### ALDH_Tt_ adopts the ALDH superfamily common structural architecture

The ALDH_Tt_ monomer is composed of the three domains common to all ALDHs (Fig. 1): (i) The NAD(P)^+^ binding domain (1–125 + 148–261) comprising a Rossman fold, (ii) the catalytic domain (267–458) and (iii) the oligomerization domain (126–147 + 494–501). (i, ii) are separated by two loops, a short linker loop region containing Glu261 (261–267), required to activate the catalytic cysteine Cys295, and a long inter-domain linker (459–493) harboring the aldehyde anchor loop (464–466). This anchor loop, which has been previously shown to contain regulatory residues such as the substrate entry channel (SEC) mouth residue^20^ and the gating aromatic residue^21^, interacts with the substrate and product. The catalytic and cofactor-binding domains form a central tunnel through the monomer with NAD(P)^+^ at one side and the classical entrance for substrate on the opposite side. The catalytic residues are deep within the center of the tunnel ~16 Å from the cofactor binding site to Glu261 and substrate entry tunnel to the catalytic Cys295. The tunnel is ~5 Å in diameter at its widest point.

### NAD(P)^+^-binding

**Figure 2:**
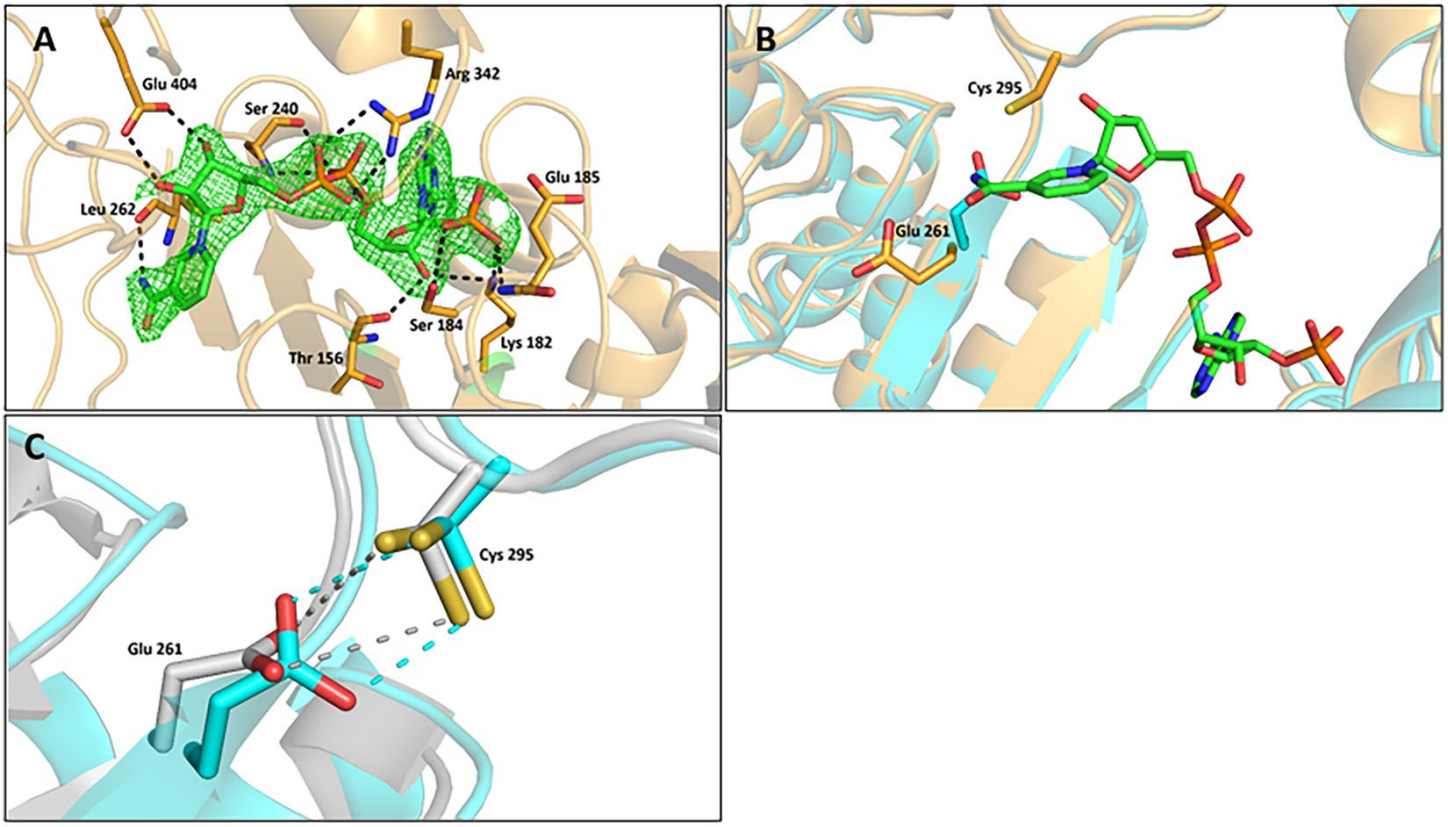
NADP^+^ binding and catalytic residues conformations in ALDH_Tt_. (**A**) NADP^+^ binding highlighting hydrogen bonding. The cofactor and interacting residues are in stick representation, the FoFc omit map of NADP^+^ contoured at 3 sigma is shown as green mesh. (**B**) Close up view of Glu261 positioning in native (orange) and ALDH_Tt_515 structures (Cyan). The catalytic cysteine 295 is shown for the native structure. The cofactor is shown to highlight the Glu261 movement induced by cofactor binding. (**C**) Superimposition of ALDH_Tt_Native and C_t_-FDH structures (PDB code2O2P) showing similarities between the two structures in terms of Glutamic acid orientation and proximity to the catalytic cysteine which is present in double conformation.

The co-crystal structure of ALDH_Tt_515 was solved with NADP^+^ bound in the expected cofactor binding cleft of the monomeric subunit. As previously observed with other ALDH structures^13,22–24^, the electron density of the nicotinamide moiety was weak compared to the ADP-ribose part of the cofactor. Similarly to the human mitochondrial ALDH and ALDH1A2^5,13^, the NADP^+^ cofactor is observed in the extended conformation in our structure. Comparison of the NADP-bound with apo structures did not reveal significant conformational changes for the adenosine pyrophosphate accommodation within the cofactor binding cleft. In the binding site the adenosine ribose interacts with Lys182 and Thr156. The 2′-phosphate group is positioned in the ceiling of the binding cleft with O2 and O3 within 3 Å distance from Glu185 carboxyl group. While these unfavorable contacts could result in electrostatic repulsion, the 2′ phosphate is stabilized by hydrogen bonds with the Glu185 backbone amide group in addition to Ser184 and Lys182 side chains. The pyrophosphate moiety is hydrogen bonded to the backbone amide group of Ser240, its side chain and to the Arg342 sidechain. Finally, the nicotinamide ribose has O2 and O3 hydrogen bonded with Glu404, while its carboxamide nitrogen is hydrogen bonded to Leu262 (Fig. 2A).

The nicotinamide ring is pointed into the catalytic pocket, in an extended “hydride transfer” conformation in both monomers A and B. The most notable difference between the NADP-bound and apo structures is observed near the catalytic residues. Indeed, in NADP-bound structures, Glu261 sidechain is in the “intermediate” conformation allowing the nicotinamide ring to sit on the bottom of the catalytic tunnel, while Cys295 is observed in the nucleophile “attacking” rotamer. This is a classical conformation associated with NAD(P)^+^ bound enzyme^23,25^. Glu261 has moved from the “In” position, where it is suitably oriented for the acylation step and the deprotonation of the catalytic thiol, to an intermediary space to allow entry of the nicotinic moiety and the transfer of a hydride from the oxyanion thiohemiacetal intermediate. However, in the native structure, Glu261 has the “In” conformation, associated with proton abstraction from the thiol prior to substrate binding (Fig. 2B). As previously observed in the human ALDH2 and C_t_-FDH structures, Glu261 is suitably oriented and within a short distance to Cys295 (Fig. 2C) to act as a general base for its activation^26–28^. Although the Glu261 side chain can be modeled correctly for the native structure, it shows a weak electron density for a buried residue. This weak electron density, as observed with other ALDHs^29^, was even absent beyond the Glu261 C_β_ of the recombinant proteins, highlighting the flexibility of this key residue. In addition, the catalytic Cys295 side chain of the native structure is observed in two different conformations, the “attacking” conformation and the “resting” conformation where the side chain is rotated away from the Glu261 (Fig. 2C). In the latter conformation, the sulfur group of the catalytic cysteine has been shown to be covalently bound to the C4 carbon of the nicotinamide ring in the case of C_t_-FDH^26^.

### A role for linker loop residues Leu262 and Gly263

**Figure 3:**
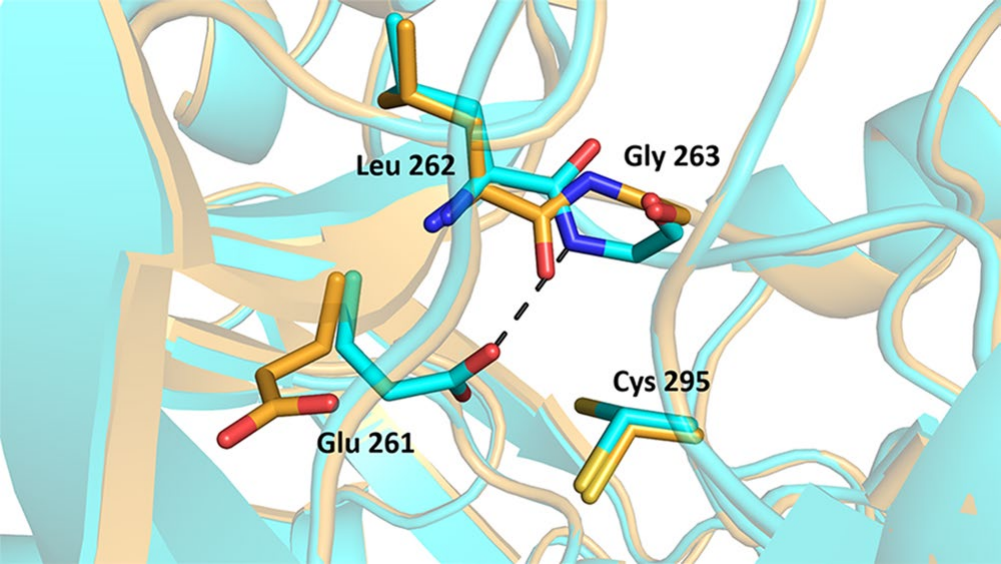
Linker loop residues orientation. Structures superimposition showing linker loop residues orientations in native (Cyan) and NADP^+^ bound (Orange) structures. The catalytic cysteine is in the attacking conformation for both structures, while the glutamic acid is in the “In” conformation for the native structure or the intermediate conformation for the NADP-bound structure. The orientation of the residues from the linker loop Leu262 and Gly263 is shown. The hydrogen bound stabilizing the glutamic acid is shown in dashes.

Besides the catalytic residues orientation upon cofactor binding, another part of the structure seems to undergo subtle movements during catalysis. Indeed, our crystal structures show differences in the orientation of Leu262 and Gly263 backbone amide and carbonyl groups. In the apo structures, the “In” orientation of Glu261 is associated with a hydrogen bonding between one of the Glu261 oxygens and the amide group of Gly263 (Fig. 3 and Supplementary Fig. S2). This interaction, when the Glu261 side chain is visible, is seen in monomer B of the apo structures. However, in monomer A and in the NADP-bound structure, a local 180° flip of the main chain bring the carbonyl group of Leu262 toward the cofactor binding site, making a hydrogen bond with the carboxamide nitrogen of the nicotinamide.

### Product release from the catalytic tunnel

**Figure 4:**
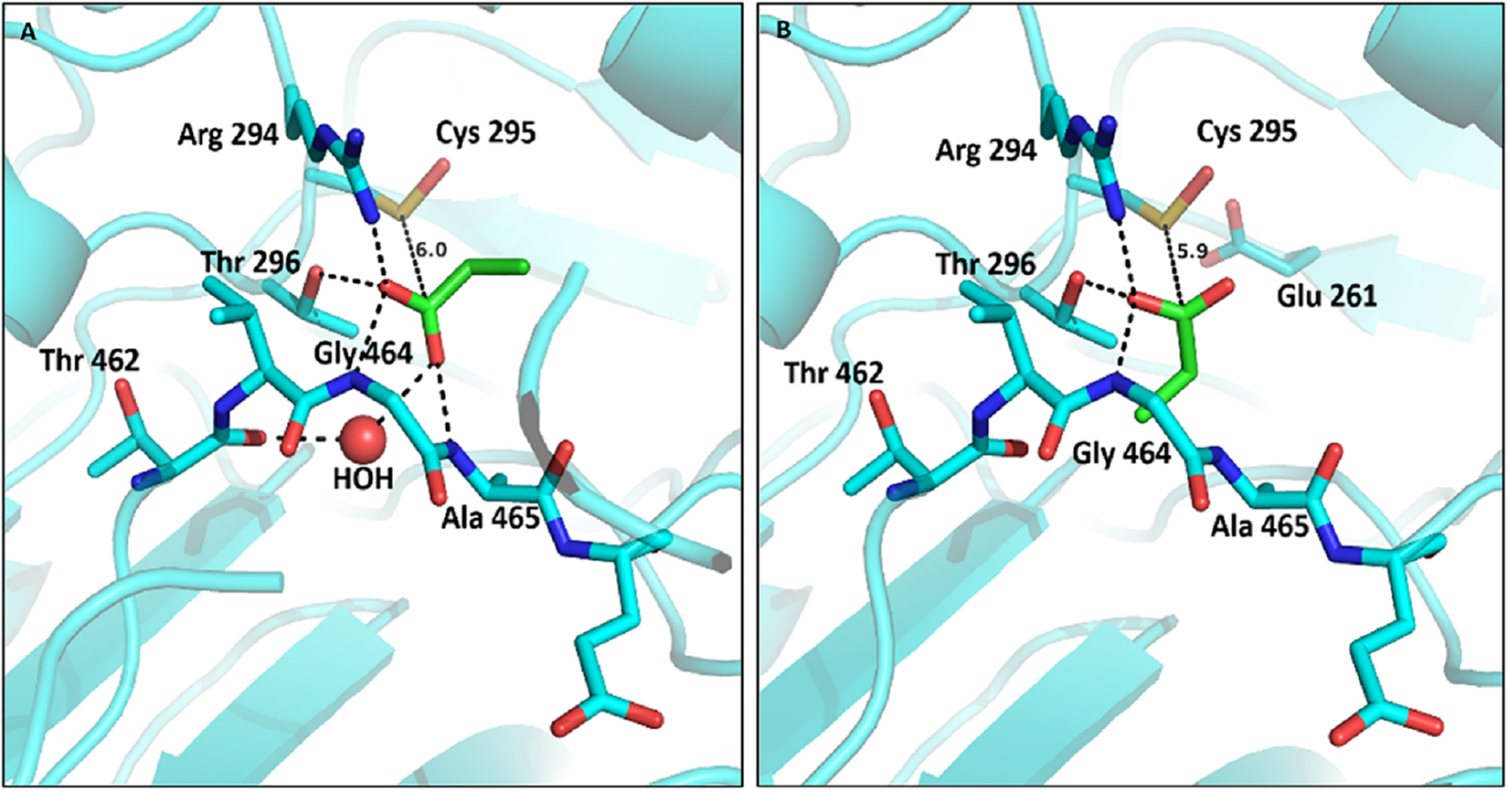
Propanoic acid in the substrate entry channel. (**A**) Product binding in monomer A of the full length recombinant ALDH_Tt_. The residues of the anchor loop (Cyan) are shown in stick and labeled. Potential hydrogen bonds between the propanoic acid (Green) and the protein residues are shown. (**B**) Product binding in monomer B, highlighting a different orientation of the carboxyl group. Glu261 orientation is shown only when the side chain was visible in the structure. The distance between the propanoic acid and the cysteine residue is shown in both structures.

The recombinant wild type crystals yielded a structure with a product molecule, propanoic acid, in monomer A and B. The product is ~6 Å from the catalytic thiol, which is in the attacking conformation, while the Glu261 can be observed in the “In” conformation in monomer B. Interestingly, the ligand, situated inside the aldehyde anchor loop within hydrogen bonding distance to Arg294, Thr296, Gly464 and Ala465, is observed in two different orientations (Fig. 4A,B and Supplementary Fig. S3). In monomer B, the carboxyl group of the propanoic acid is oriented toward the catalytic cysteine and is coordinated by hydrogen bonds with Arg294, Thr296 and Gly464. However, in monomer A the carboxyl group of the product is now oriented toward the aldehyde anchor loop and coordinated by the same residues as in monomer B in addition to Ala465 and Thr462 through a water-mediated hydrogen bond.

### C-terminal arm interactions and significance

**Figure 5:**
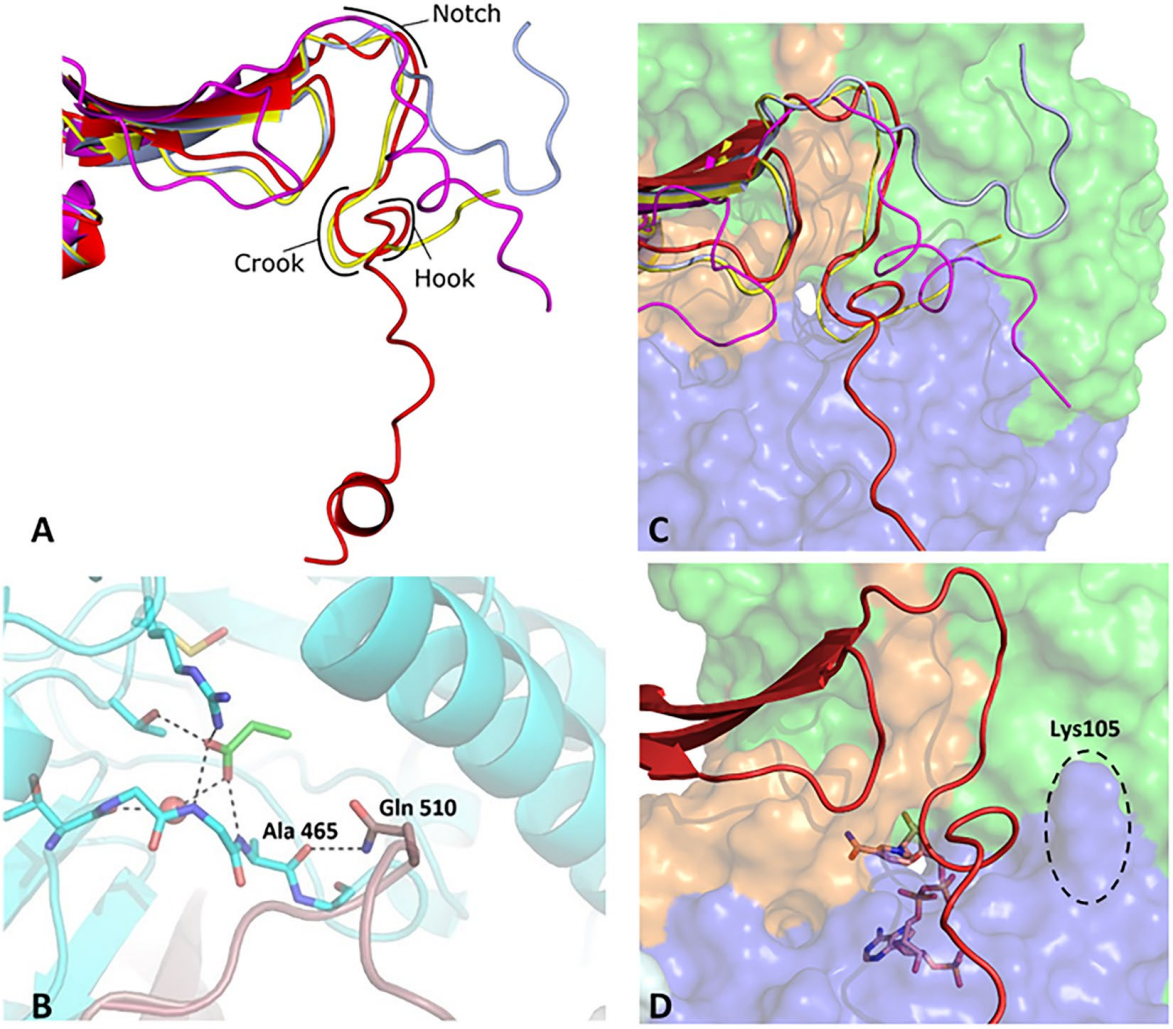
Comparison of ALDH tail orientations with regard to the substrate entry tunnel and fulcrum points on the tails. (**A**) Superimposition of ALDH_Tt_ (Red), Human dimeric ALDH3 (PDB: 3SZA, magenta) and tetrameric ALDH7A1 in the closed and open conformations (PDB: 4ZUL, yellow; 4ZUK, light blue respectively). Highlights how all previously characterized tails fold at the crook or notch and rest in the interdomain cleft (domain coloring of Fig. 1 used) whilst ALDH_Tt_ is orientated across the tetramer interface and away from the other tails after the hook. (**B**) Close up of the substrate entry channel showing interaction between the C-terminal tail Gln510 of monomer B (brown) with Ala 465 of monomer A (Cyan) in ALDH_Tt_. (**C**) Comparison of human ALDH7A1 and ALDH_Tt_. Domain coloring is kept consistent with Fig. 1 for orientation purposes. The human dehydrogenase in the closed position can swing out away from the substrate entrance channel into the open conformation and fold into the interdomain cleft and against the catalytic domain due to its size whilst ALDH_Tt_’s extended tail makes this impractical. (**D**) A magnified view of ALDH_Tt_ SEC is shown highlighting the positively charged K105 packing against the negatively charged hook of the tail and not allowing the tail to fold like those previously characterized. The crook is seen to be firmly over the SEC. NADP^+^ is depicted in stick within the tunnel.

The tail of ALDH_Tt_ starts after the oligomerization domain and terminates in a 3-turn helix, which interacts with the N-terminal residues of its opposing monomer (A-D). The novel orientation of the tail contributes to salt bridge formation and hydrogen bonds between monomers A/B and A/D. This is governed by three pivot or fulcrum points on the tail (Fig. 5). In the available structures on the PDB, ALDHs with extended C-terminal are observed; however, their tails are sensibly shorter and do not interact with N-terminal residues of the other monomers (Supplementary Fig. S4).

The ALDH_Tt_ tail begins in the cleft between the catalytic and cofactor binding domains. It makes an immediate turn at Ser503 and Gly504 (referred to as the “Notch” in Fig. 5A) and deviates from the well-studied dimeric tail folding orientation wrapping with the N-terminal residues of another monomer. This sharp turn makes the tail run perpendicular to the direction of the inter-domain cleft and directly over the substrate access tunnel. The section of the tail blocking the SEC mouth is known as the “Crook” (Fig. 5A). In ALDH_Tt_ this pivot is made possible through interactions between Leu508 main chain and Asp512 amine group and side chain. The SEC is tightly locked by the side chains of Ala509 and Gln510 pointing into the pore. Gln510 forms a hydrogen bond with Ala465 on the aldehyde anchor loop (Fig. 5B). This interaction suggests that the tail plays a role in active site regulation^30^.

The ALDH7A1 tail is far shorter than its ALDH_Tt_ counterpart and has adequate space to make a 16 Å sweep away from the tunnel entrance (Fig. 5C). The movement occurs at the “Notch” region which is characterized by the presence of asparagine and serine residues (Glu501 and Ser503 in ALDH_Tt_) interacting together via a hydrogen bond. In ALDH_Tt_ an additional salt bridge between Gln507 and Glu501 further stabilizes the “Notch” region. The current ALDH_Tt_ structures do not indicate that this movement is possible due to the increased length and differing orientation. The tail does not make the distinctive “L” shape reported for ALDH7A1. It rather deviates again in the “Hook” region creating a straight line path from oligomerisation domain to the N-terminus of a neighboring monomer. The “Crook” of the tail (Leu508-Gln510) is conserved in both structures as well as the interacting anchor loop residues (Gly464-Glu466). This conservation is thought to be a defining characteristic of family 7 of the ALDH superfamily, so it is possible that ALDH_Tt_ should be classified in family 7. In all ALDH_Tt_ structures, where the tail is persistently closed, the anchor loop is also closed. The ALDH7A1 structure terminates four residues after the “Crook” allowing room for the tail to fold into a hydrophobic pocket in the side of the catalytic domain. However, the ALDH_Tt_ terminates twenty residues after the “Crook” leaving insufficient space for a long tail to fold away from the substrate entrance tunnel. This swinging of the tail into an open position is unlikely in ALDH_Tt_, due to Lys105 pointing up into the interdomain cleft (Figs 1 and 5D).

### Salt bridge mediated tail-oligomerization domain interactions

The wrapping of the tail around the outside of the tetramer, via the “Hook”, and blockage of the substrate access tunnel via the “Crook”, is mediated by a network of hydrogen bonds and salt bridges between the oligomerisation domains and the tails. These interactions keep the entire tail pressed against the monomer interfaces (A-B-D), strengthening them and making it far stronger than mesophilic counterparts. The linchpin of these interactions revolves around an additional salt bridge in the oligomerisation domain of each monomer, Arg126-Glu136. The Arg126 of monomer D points toward the pivotal tail fulcrums and creates a central interaction point between the “Crook” and “Hook” on the tail of monomer A, the anchor loop of monomer B and the oligomerisation domain of monomer D. The Arg126-Glu136 is thought to be the strongest of salt bridges^31^, in other ALDHs the arginine is replaced with an aspartic acid incapable of forming a salt bridge with the negatively charged glutamic acid. This salt bridge stabilizes the tail of each monomer by mediating hydrogen bonding between Glu136 and Gln510, which holds the tail in place over the substrate access tunnel. The rest of the tail is characterized by the presence of three proline residues in positions 518, 521 and 523, hindering the formation of any secondary structures. Finally, the tail is held on the surface and interacts with the N-terminal of monomer D through hydrogen bonds involving main chain amine and carbonyl groups. A summary of these interactions is shown in Supplementary Fig. S5.

To probe the significance of the tail and its fulcrum points, two truncated tail mutants were made: (i) ALDH_Tt_515 which removes all residues distal to the “Crook & Hook” and (ii) ALDH_Tt_508, a mutant which removes the “Crook & Hook” entirely but retains the “Notch” to try and model other tail containing ALDHs. It was expected that at least one of the truncated mutants of ALDH_Tt_ could produce an open conformation of the protein and obtain substrate/product bound structures. However, ALDH_Tt_ mutants could only be crystalized in a closed and compact conformation. ALDH_Tt_515 demonstrates no movement in the anchor loop or salt bridge interactions, leading us to believe that the last 15 residues of the protein play a minor role in the active site regulation. The ALDH_Tt_508 structure is “open” as the “Crook” of the tail has been removed, but there is still no movement in the anchor loop to an open position. It is worthy to note that the electron density maps were of a reduced quality in the C-terminal region of the truncated mutants compared to the native or the full length recombinant proteins, highlighting the role of the extended C-terminal tail in stabilizing the overall structure.

Comparison with homologous sequences (Table 1) shows that the *T*. *thermophilus* HB8 strain is the only one with a significantly shorter tail within its genus (515 *vs*. 530 residues). Within the *Deinococcus*-*Thermus* phylum, however, not all are thermophilic bacteria. A careful DELTA-BLAST search against all structures in the PDB indicated the absence of a similar long C-terminal tail. Nonetheless, this tail is present not only within the *Deinococcus-Thermus* phylum (Table 1), but also in several other organisms including *Bacillus stratosphericus* (GenBank: EMI11952.1; 539 residues), *Pyrinomonas methylaliphatogenes* (GenBank: CDM65952.1; 545 residues) and *Thermaerobacter marianensis* (GenBank: ADU51624.1; 552 residues). Despite a similar extensive search using only the C-terminal tail sequence (EYSGRLQLAQMDTGYVSPKAPTPWGEVLGL) against protein sequences outside the *Deinococcus-Thermus*phylum, only a partial sequence from *Kouleothrix aurantiaca* (GenBank: KPV51990.1; a member of the Chloroflexi phylum of filamentous green non-sulfur bacteria) was identified as containing the fragment SGKLQLAQMDTDYIAPKSP.

**Table 1:** Distribution of ALDH with long C-terminal tail.

Clade ID	Organism	Accession	Locus_tag	Length (aa)
20358	*Deinococcus proteolyticus MRP*	WP_013623073	Deipr_2214	539
20699	*Holophaga foetida DSM 6591*	WP_005036551	HolfoDRAFT_1243	537
20356	*Marinithermus hydrothermalis DSM 14884*	WP_013704042	Marky_1255	533
20349	*Meiothermus ruber DSM 1279*	WP_013013654	Mrub_1373	529
20350	*Meiothermus silvanus DSM 9946*	WP_013158503	Mesil_2080	530
21980	*Meiothermus timidus DSM 17022*	WP_018465841	B047_04905	535
20355	*Oceanithermus profundus DSM 14977*	WP_013457786	Ocepr_1159	530
20352	*Thermus aquaticus Y51MC23*	WP_003045659	TaqDRAFT_5353	530
22722	*Thermus igniterrae ATCC 700962*	WP_018112318	B128_10945	530
21982	*Thermus oshimai DSM 12092*	WP_018460723	B043_01690	530
21982	*Thermus oshimai JL-2*	WP_016328881	Theos_0625	530
20353	*Thermus scotoductus DSM 8553*	WP_019550178	F604_01840	529
20353	*Thermus scotoductus SA-01*	WP_015717232	TSC_c13390	530
20354	*Thermus sp*. *CCB_US3_UF1*	WP_014515534	TCCBUS3UF1_11310	530
20351	*Thermus thermophilus HB27*	WP_011172958	TTC0513	530
20351	*Thermus thermophilus HB8*	WP_011228252	TTHA0865	515
20351	*Thermus thermophilus JL-18*	WP_014629714	TtJL18_1181	529
20351	*Thermus thermophilus SG0*.*5JP17-16*	WP_014510213	Ththe16_0870	530
20357	*Truepera radiovictrix DSM 17093*	WP_013178702	Trad_2228	527

All species are within the *Deinococcus*-*Thermus* phylum with the exception of *Holophaga foetida* which is a member of the *Acidobacteria* phylum. Only the ALDH from *T*. *thermophilus* HB8 has a significantly shorter C-terminus.

### The central pore of the tetramer

**Figure 6:**
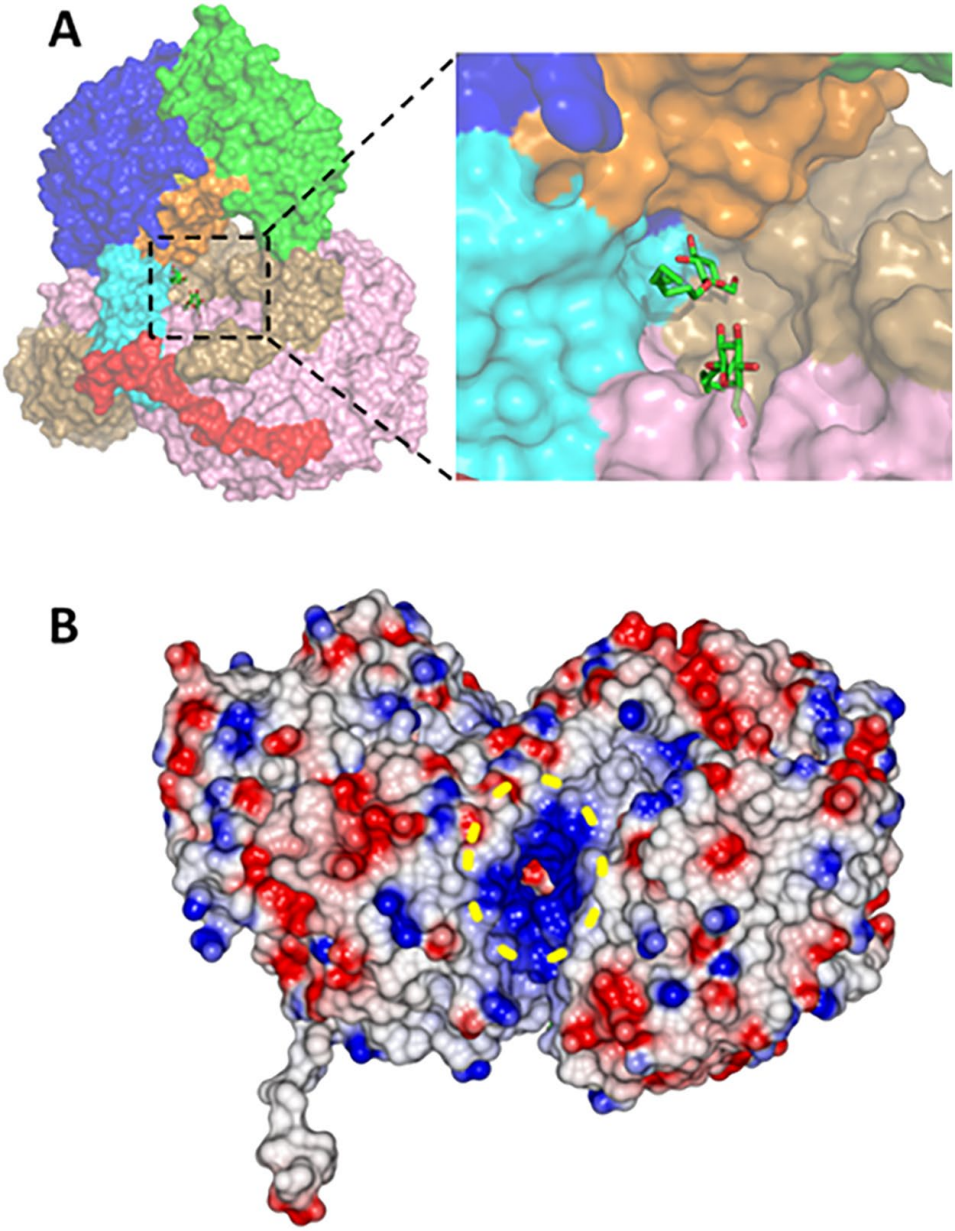
Positioning of BOG in the native structure deep within the tetramer. (**A**) Surface representation of the tetramer in which monomer B has been removed for clarity purposes. The two BOG molecules (in stick representation) are deeply buried within the tetramer. Note that BOG molecules lie between the inter-domain linker and the oligomerization domain (Orange and cyan, respectively for monomer A). Coloring for all monomers is kept consistent with Fig. 1 to aid orientation. (**B**) Yellow dashed ellipse highlights the highly positive tunnel which exists between monomer interfaces.

In the native crystal structure two partially occupied and mutually exclusive *n*-octyl-β-glucoside (BOG) molecules originating from the detergent used for *caa*_3_-oxidase purification; (cf. Materials and Methods) are found deep within the centre of the tetramer. They lie in a highly positive pore created by the oligomerisation domain and inter-domain linkers of each monomer. Pores and tunnels also exist between monomer interfaces in the tetrameric assembly and these interfaces are highly positively charged in the ALDH_Tt_ structure, far more so than many other known ALDH structures (Fig. 6 and Supplementary Fig. S6).

In the tetramer, there are four tunnels leading to this central pore. The tunnel openings consist of residues from the cofactor binding domain of one monomer and residues from the interdomain linker and catalytic domain of another. These tunnels are again ~15 Å in length before reaching a central junction and accessing a shared tunnel leading to the pore which is ~25 Å in length. The maximum diameter of these tunnels is similar to the substrate access tunnel, 3.0–3.2 Å.

The central cavity is composed of residues from the interdomain linker, oligomerisation domain and the C-terminal tail. There is hydrogen bonding between the head group of the BOG and the positively charged sidechain of Arg127 on the junction between the oligomerisation domain and cofactor binding domain which is positioned directly beside Arg126 which we believe to play a crucial role in tail orientation and thus active site access.

### Enzyme kinetics

As the cofactor used in any enzyme assay has a significant effect on its readout, we first determined the preferential cofactor using a set of substrates for the recombinant wild type enzyme ALDH_Tt_530. The ALDH activity was consistently higher with NAD^+^ with a specific activity of 0.965.8 ± 0.043 U mg^−1^ compared to with NADP^+^ at 0.322 ± 0.059 U mg^−1^ at 50 °C (Supplementary Table S1). Secondly, we also defined the optimal pH value for the assay, within a range of pH 6.0–8.0 using Kp_i_ buffer with the highest activity being at pH 8.0 (data not shown). Further assays were performed at both mesophilic temperature (25 °C) and elevated thermophilic-like temperature (50 °C). Although *T*. *thermophilus* grows best at 70 °C, a lower temperature was used in the assays to minimize evaporation of certain volatile substrates and maintain stable readings.

Interestingly, both truncated mutants ALDH_Tt_508 and ALDH_Tt_515 were slightly more active (specific activity of 1.068 ± 0.004 and 1.069 ± 0.012 U mg^−1^, respectively, Supplementary Table S1) than the recombinant wild type enzyme at 50 °C (0.965 ± 0.043 U mg^−1^), perhaps due to the presence of small amounts of ammonium sulfate acting as a stabilizing agent. Attempts to assess enzymatic activities at the higher temperature of 85 °C were not successful as both mutants rapidly lost their function compared to ALDH_Tt_530 where it was still active up to 4 min, consistent with the Tm of ALDH_Tt_530 of 84 °C. Taken together, the relative stability of the recombinant enzymes was in the order ALDH_Tt_530 > ALDH_Tt_515 ≈ ALDH_Tt_508 (Supplementary Fig. S7).

## Discussion

The structures described here show a unique tetrameric organization for ALDH. Indeed, ALDHs are known to form dimer-of-dimers tetramer. However, the presence in ALDH_Tt_ of an unusual extended arm that wrap the facing monomer through H-bonds and salt bridges shows for the first time how the tetramer is not merely a dimer-of-dimers carrying out their functions independent of each other, but that the interaction of the N- and C-termini in CS-related monomers may regulate active site access in NCS-related molecules.

ALDHs are NAD(P)^+^ dependent enzymes. Structurally, the NAD(P)^+^ binding sites do not belong to a single common family as classified in^32^ with ALDH_Tt_ situated in Group III_NADP family. Due to the poor discriminatory power of this family, it is not possible to state whether the enzyme binds to NAD^+^ and/or NADP^+^ on the basis of sequence alone. Despite the co-crystals of the truncated mutant ALDH_Tt_515-NADP^+^ being significantly larger and better diffracting than those with NAD^+^, enzyme kinetics data described in this work indicates that the preferential cofactor is NAD^+^. In addition, these data are supported by the observed proximity of the 2′ phosphate to Glu185, which gives the structural basis for NAD^+^ preference in ALDHTt.

The structure of ALDH_Tt_ with NADP^+^ shows no major conformational changes between the apo and cofactor-bound structures. The effect of NADP^+^ binding is observed locally on the catalytic residues Glu261 and Cys295 side chains which adopt classical orientations observed with cofactor bounded ALDH^23,25^. It is worthy to note here that the electron density of the nicotinamide moiety was weaker than the rest of the cofactor. This is mainly due to the high flexibility of this part of the cofactor, thought to sample different conformations during catalysis^13,33–35^.

Besides moving Glu261 away from the “In” position, cofactor binding induces more subtle changes in the active site. This concerns a 180° main chain flip around residues of the linker loop Leu262 and Gly263 which interact with Glu261 and the nicotinamide moiety of the cofactor, respectively. These kinds of interactions between the linker loop and the nicotinamide moiety, as previously reported for C_t_-FDH, are believed to play a role in the cofactor stabilization^26^. Consequently, these movements are likely to play a role during catalysis, as they involve direct interactions with the general base Glu261 and the nicotinamide moiety of the cofactor. As for ALDH2, the proximity of Glu261 with Cys295 argues for a direct activation of the catalytic cysteine by the glutamic acid. The latter, being in the “In” conformation, is stabilized by a hydrogen bond with Gly263. Upon cofactor binding, Glu261 rolls back to make a place for the cofactor, this movement is accompanied by a local 180° flip of the main chain linker loop to bring Leu262 carbonyl group toward the carboxamide nitrogen of the nicotinamide for stabilization. This will allow for the hybride transfer to occur, and for another 180 ° flip of the main chain linker loop along with “In” positioning of Glu261 following cofactor exit. This last step is supported by our product-bound structure where the “In” conformation of Glu261 is observed. Indeed, in the full-length recombinant protein Glu261 is observed in the “In” conformation, which may be indicative of an intermediate state depicting the molecule leaving the tunnel after catalysis, with repositioning of the glutamic acid. The different orientations of the product in monomers A and B is likely to provide snapshots of product handling throughout the aldehyde anchor loop. It is worthy to note that a similar product orientation was previously observed with ALDH1A3^36^.

Initially, when we solved the structure of the native ALDH_Tt_, we noticed the presence of an extended C-terminal tail. This is not surprising as similar extensions have been seen in the *ba*_3_-oxidase, *caa*_3_-oxidase and cytochrome *c*_552_ of *T*. *thermophilus* previously. In contrast, thermophilic targets are expected to have shorter loops and tighter packing^37–39^.

C-terminal tails are not uncommon in ALDHs but are most often characterized in dimeric ALDH^40^, with the exception of the human tetrameric ALDH7A1^30^. However, in all previously reported cases, this tail is of a considerably shorter length and does not interact with the N-terminus of a CS-related molecule. To the best of our knowledge, all known tails only interact with the closest monomer in the classical dimer orientation (A/B). An interaction between N- and C- termini is not possible due to the fact that dimeric ALDHs have a shortened N-terminus and tetrameric ALDHs do not contain a tail.

Two structures in the PDB show evidence for the tail of an ALDH being involved in active site access and regulation. These are membrane associated human FALDH^41^ (PDB: 4QGK) and ALDH7A1^30^ (PDB: 4ZUL). The FALDH contains a transmembrane helix (omitted from structure) and a gatekeeper helix which does not plug the active site entrance but merely caps it. ALDH7A1 is more similar in structure to ALDH_Tt_ as the “Crook” of the C-terminal tail plugs the substrate entrance tunnel firmly. Although dimeric ALDH possess a tail, it should be noted that in many cases the tail is not long enough to cap or plug the substrate access tunnel. In cases where it is long enough, it folds along the edge of the tunnel opening without ever interacting with the substrate entrance channel mouth residues.

In all tail containing ALDHs characterized thus far, the apo and cofactor bound proteins were in an open conformation. A closed conformation could only be found in product bound ALDH7A1. Surprisingly, ALDH_Tt_ could only be crystallized in a closed tail conformation in the apo and holoenzyme. The difference in resting conformations between ALDH_Tt_ and other ALDHs may be indicative of an allosteric regulation mechanism such as the model seen for non-phosphorylating GAPN^16^ or the requirement of stabilizing ligands between monomer interfaces^42^.

The longer tails in ALDHs were postulated to effect solubility in medically significant mutations such as c.1512delG which leads to pyridoxine-dependent epilepsy^30,43^. We would like to note that our truncated mutants were of a much lower solubility than full length recombinant or native proteins highlighting the significance of the N- and C-termini interactions in the quaternary structure. We also investigated the tail extension as an obvious thermal clamp in a thermophilic genus such as *Thermus* however, no significant reduction in T_m_ (<5 °C) was seen during thermal shift assays. This is unsurprising when it is noted that an ALDH of 515 residues in length exists in the close relative, *T*. *thermophilus* HB8. Such a protein would still contain a full complement of “Crook & Hook” tail residues allowing for active site regulation and may even perform more efficiently as it may be able to fold more easily into inter-domain clefts without the cumbersome longer tail.

The similarity in tail orientation and active site regulatory loops between ALDH_Tt_ and its deletion mutant structures even without intermolecular interactions between the end of the tail and N-terminal interactions hints that active site access is regulated by additional domain interactions. It must be noted that the similar crystallization conditions and thus uniform space group may only allow for the closed and compact conformation of the protein. Phylogenetically, we hypothesize that the ancestral ALDH possessed both extended N- and C-termini, but either terminus was lost due to no evolutionary pressure being present in accordance with the potentially cumbersome regulation model needed in ALDH_Tt_. Our structural data shows for the first time a clear rationale highlighting that ALDH first evolved with both extended N-termini and then diverged as either terminus was truncated.

The presence of longer tails in mesophilic bacteria belonging to the *Deinococcus-Thermus* phylum suggests that the presence of this C-terminal tail must have been chosen by evolution for specific functional reasons not, entirely, due to its habitat of high temperature. The very limited number of sequences with an extended c-terminal tail found outside the *Deinococcus-Thermus* phylum may argue for a phylum-specific adaptation that could have been subsequently lost over the course of evolution, especially considering that this phylum is rather ancient.

The oligomeric state of ALDH poses an obvious question as to the reason for the conservation of a tetrameric assembly even when the protomers are far apart in the three-dimensional so as to preclude any direct electron transfer (*cf*. for instance, ubiquinone:cytochrome*c* oxidoreductase/*bc*_1_ complex^44^). Consequently, an alternative explanation must be considered – the extensive contacts made by the oligomerization domain potentially provide a structural stability even when the living conditions are not extreme. In this regard, the ‘ancient’ tetrameric assembly was not lost when this enzyme was retained in human and other mesophilic organisms.

For human ALDH, the C-terminal arm confers only a stability enhancement but is not an oligomerization-triggering element. This is the reason that the deletion of the ALDH3 tail, addition of various tails in ALDH1, and mutations at the dimer-dimer interface failed to convert one natural state to another^45^, in line with previous observations that the protein stability and folding are effected by the N-terminal residues^46,47^. Nonetheless, given the magnitude of free energy change upon tetramerization, we could not isolate a dimeric variant of ALDH_Tt_ in a pH- and cofactor-dependent manner in contrast to the horse liver enzyme^3,12^.

Another intriguing observation with ALDH_Tt_ is the presence of BOG molecules at the dimer-dimer interface. These BOG molecules have been modelled at partial occupancy which suggests that the detergent molecules sample several conformations in the tetramer’s pore. BOG has been used previously to study the cornea crystallin ALDH and was noted as being mild and non-interfering with regards to the quaternary structure^48,49^. The adventitious binding of detergents as additives in soluble proteins or as lipid mimics in membrane protein crystallisation is not uncommon and often is overlooked as having any biological significance. However, the presence of BOG deep within the tetramer assembly of ALDH_Tt_ is interesting as its positioning, with respect to the rather inflexible and closed position of the tail, begs the question as to how it entered

It could hardly be suggested that the BOG molecules entered the central pore of the tetramer *via* one of the tunnels present in the structure. Indeed, these tunnels are not of a sufficient size to allow the bulky glucose head group of a BOG to enter and make its way to the central pore, so an alternative conformation or access pathway must exist. Another possibility is that: (1) the tail swings away from the wall of the central pore attempting to allow substrate to enter into the monomeric subunit (2) the BOG cannot fit into the monomer so continues into the positively charged pore and (3) the tail closes again sequestering the detergent inside. One final possibility may be that the detergent has split the tetramer into dimers; however, this seems unlikely as there is no presence of detergent molecules on the other surfaces of the protein and BOG has been shown to be mild towards ALDH in the past. The presence of two conformations of detergent molecule most likely points to the two positions of the molecules when they enter from opposing interface tunnels. The negative head group of the detergent molecule is attracted to this charged pore and then becomes trapped in the central cavity. It is tempting to postulate that this highly charged pore is not coincidental and that this is a possible allosteric regulation site for ALDH_Tt_ which allows access to the substrate tunnel of each monomer.

Interestingly, the truncated mutants were kinetically more active than the full-length ALDH_Tt_. The occlusion of the active site by the C-terminal arm in ALDH_Tt_530 could be the reason for the increased activity in the truncated enzymes but the crook and hook fulcrums are still present in ALDH_Tt_515 so this was expected to also have a similar activity to the recombinant wild type. The higher activity in the truncations may be explained by the easier folding of the tail away from the SEC like other ALDH discussed above but our structural data contradicts the movement. It may be the case that a folded or open crook and hook portion of the tail may exist during the *in vitro* assay, but the current crystallization conditions do not stabilize such a conformation during structural studies. Regardless, this higher activity is not observed at the higher temperature of 85 °C as both mutants rapidly lost their function compared to ALDH_Tt_530 where it was still active up to 4 min, consistent with the Tm of ALDH_Tt_530 of 84 °C.

Extrapolating our data herein to other previously-characterized enzymes is not possible as mesophiles and thermophiles have different optimal temperature. Another reason for a reserved comparison of ALDH is that the true substrate for many has not been identified and as such cannot be compared with ALDH kinetic data for enzymes with their actual physiological substrate defined. For instance, the extensively-studied *E*. *coli* aldehyde dehydrogenase, AldB, has a *K*_M_ of 5.8 µM with the substrate propanal^42^, compared to the ALDH_Tt_530 *K*_M_ of 2.76 mM (data not shown). Perhaps a more straightforward comparison would be with the enzyme from *T*. *thermophilus* HB8.

An ALDH in *T*. *thermophilus* HB8, which forms a complex with an aldolase and occupies an interesting subclass of the ALDH superfamily^50^ also had its kinetic parameters defined at 25 °C with a *K*_M_ = 6.4 mM compared to the ALDH_Tt_530 *K*_M_ = 2.76 mM when propanal was used. However, a much more kinetically active enzyme exists in another thermophilic bacteria, *Geobacillus thermodenitrificans*, which is reported as having a *K*_M_ of 6.6 µM using acetaldehyde at 60 °C^14^. Again, this increased kinetic ability may be due to the higher temperature but when attempted at 60 °C we could not obtain reliable data due to the volatile nature of the aldehydes.

## Materials and Methods

### Native ALDH_Tt_ protein purification and crystallization

Crystalline native ALDH from *T*. *thermophilus* was identified as an impurity, via Edman degradation (deformylated) and mass spectrometry, during the crystallization of the *caa*_*3*_-cytochrome *c* oxidase whereby the oxidase and ALDH_Tt_ crystals grew in the presence of polyethylene glycol and ammonium sulfate as precipitating agents, respectively^18^. ALDH_Tt_ was purified from the *caa*_*3*_-oxidase by cation exchange chromatography and ammonium sulfate precipitation. Suitable bipyramidal crystals from 20 mg/ml solution were obtained by the sitting-drop vapor diffusion method at 20 °C, against 50 mM MOPS pH 7.5 and 1.2 M ammonium sulfate.

### Cloning, production and crystallisation of wild type recombinant ALDH_Tt_ and its truncated mutants

Primers used for amplification and cloning of the gene encoding for ALDH_Tt_ (TTC0513; Accession ID: WP_011172958) into the pET-22b (+) vector are summarized in Supplementary Table S2.

The construct DNA for each of the wild type and truncated mutants of ALDH_Tt_ were transformed into *Escherichia coli* BL21 Star (DE3) competent cells (Invitrogen). The auto-induction ZYM-5052 medium^51^, supplemented with ampicillin (100 µg/ml), was inoculated with an overnight culture of the transformed cells at 1% (v/v), and grown for 48 h at 25 °C with shaking at 200 rpm.

Cells were collected by centrifugation (6500 × *g*, 15 minutes, 4 °C) and washed in 500 ml of 20 mM Tris-HCl pH 7.5, 5 mM β-mercaptoethanol, 10 mM imidazole and 500 mM NaCl. Following a second centrifugation step, the cells were resuspended in the same buffer supplemented with Lysozyme (1 mg/ml) and 5 mM EDTA pH 8.0 and stirred at room temperature for 1 h. DNase (0.1 mg/ml) was then added along with 5 mM MgCl_2_ and the cells were gently stirred at 4 °C for 1 h and sonicated in an ice-water bath for 20 min. Finally, the mixture was heated to 65 °C for 15 min and centrifuged at 25,000 × *g*, 30 min, 4 °C). The supernatant was then loaded onto an XK 16/20 column containing Ni Sepharose 6 Fast Flow (GE Healthcare) pre-equilibrated with 20 mM Tris-HCl pH 7.5, 5 mM β-mercaptoethanol, 10 mM imidazole and 200 mM NaCl. Bound proteins were eluted using a step gradient of 60, 100, 250 and 500 mM of imidazole in 20 mM Tris-HCl, 5 mM β-mercaptoethanol and 150 mM NaCl, pH 7.5. Fractions containing the protein were then dialyzed overnight in 50 mM Tris-HCl, 5 mM β-mercaptoethanol and 250 mM NaCl, pH 7.5 prior to concentration using Amicon Ultra-15 centrifugal filters, 50 kDa MWCO (Merck Millipore) and loaded onto a HiLoad 16/60 Superdex 200 pg column pre-equilibrated with 50 mM Tris-HCl pH 7.5, 5 mM β-mercaptoethanol and 150 mM NaCl.

All crystallization assays were realized in the same conditions as for native ALDH_Tt_. Co-crystallisation of ALDH_Tt_515 with NAD(P)^+^ and ALDH_Tt_530 with propanal were achieved by adding 1 mM of NAD(P)^+^ sodium salts and 50 mM of propanal to the respective drops.

### Structure solution of ALDH_Tt_ native and recombinant proteins

Datasets were collected at the Diamond I04 and I24 beamlines. Data were processed using xia2/XDS^52–54^ and high resolution cut off were selected according to Karplus & Diederichs^55^. For ALDH_Tt_native, 6 X-ray diffraction data were collected from multiple crystals, each having different effective maximum individual resolution as determined by the CC_1/2_ at the CORRECT stage. With the availability of multiple highly-diffracting isomorphous crystals, it was possible to merge and scale the datasets using XSCALE into a single dataset. Phasing was achieved through molecular replacement (phenix.MRage/Phaser)^56^ using multiple search models. The best solution was obtained with the human ALDH1A1 (PDB ID: 4WJ9)^57^ in the space group *P*4_1_2_1_2. The partial model was automatically built using Phenix.AutoBuild^58^, improved manually with Coot^59^ and refined with phenix.refine^60^ and Refmac^61^. The obtained model was used to resolve the structure of the recombinant proteins. Phenix.Ligandfit was used to automatically place ligands in the structures. Data collection and refinement statistics are shown in Supplementary Table S3.

### Thermofluor assay

A thermofluor assay was used to assess the relative thermostability of the ALDH_Tt_ samples. The dye Sypro orange (Invitrogen) and a qPCR machine (Roche Light cycler 480) with an excitation filter of 498 nm and an emission filter of 610 nm were used. Samples were equilibrated at 25 °C for 3 min before a ramped heating step between 25–95 °C. The heating rate was set to 0.03 °C/s corresponding to 20 acquisitions/°C. The first derivative and T_m_ were plotted using OriginPro 9.1.

### Enzyme kinetics

Enzyme assays were performed over a short period of time to ensure no evaporative loss of the volatile aldehydes. The standard assay condition was chosen and optimized from the conditions which gave the largest and most stable change in the fluorescence readings. The standard enzyme assay consisted of 10 mM KH_2_PO_4_-K_2_HPO_4_ (Kp_i_) pH 8.0, 0.4 mM NAD^+^, 60 nM ALDH, 1 mM hexanal, in a total volume of 0.5 ml unless otherwise stated. A substrate-lacking reaction was used as a negative control. The reaction was monitored at 50 °C for 60 s. The reduction of NAD^+^ cofactor to NADH was monitored in a Cary fluorescence spectrophotometer (λ_excitation_ = 340 nm; λ_emission_ = 463 nm) with respective slit widths of 20 nm and 5 nm. All components were heated before being added to the cuvette and the reaction was started with the addition of substrate. One unit of enzyme was defined as the amount of enzyme which catalyzed the formation of 1.0 µmol of NADH/min. All assays were performed as a minimum of two independent experiments with triplicates for each reaction. Kinetics data and curve fitting were performed using OriginPro 9.1.

### Figure generation and data deposition

All the figures were generated with Pymol (DeLano Scientific, South San Francisco, CA) and CCP4mg^62^. The atomic coordinates and structure factors for ALDH_Tt_ native, ALDH_Tt_530 in complex with propanoic acid, ALDH_Tt_515 in complex with NADP and ALDH_Tt_508 have been deposited in the Protein Data Bank www.pdb.org (PDB ID: 6FJX, 6FK3, 6FKU and 6FKV, respectively).

## Electronic supplementary material

Supplementary information
