# Supplementary material for: The quaternary structure of Thermus thermophilus aldehyde dehydrogenase is stabilized by an evolutionary distinct C-terminal arm extension
Source: Sci Rep. 2018 Sep 6;8:13327. doi: 10.1038/s41598-018-31724-8 (PMC6127216; doi:10.1038/s41598-018-31724-8)
Supplement: Supplementary file 1 — Supplementary information [file 41598_2018_31724_MOESM1_ESM.pdf]

The quaternary structure of *Thermus thermophilus* aldehyde dehydrogenase is stabilized by an evolutionary distinct C-terminal arm extension

Kevin Hayes[1,2], Mohamed Noor[1,2], Ahmed Djeghader[1,2], Patricia Armshaw[1,2], Tony Pembroke[1,2], Syed Tofail[2,3], Tewfik Soulimane[1,2,*]

[1] Department of Chemical Sciences, University of Limerick, Limerick, V94 T9PX, IRELAND

[2] Bernal Institute, University of Limerick, Limerick, V94 T9PX, IRELAND

[3] Physics Department, University of Limerick, Limerick, V94 T9PX, IRELAND

[*] Corresponding author: Tewfik.Soulimane@ul.ie
Tel: +353-61-234133
Fax: +353-61-202568

Supplementary tables and figures

Supplementary Table S1. Kinetic properties of $ALDH_{Tt}530$ and truncations mutants at 50 °C.

	V_{max} (U mg^{-1})	K_{cat} (S^{-1})
**$ALDH_{Tt}530$ (NAD^+)**	**0.965±0.043**	**0.942±0.042**
**$ALDH_{Tt}530$ ($NADP^+$)**	**0.322±0.059**	**0.314±0.058**
**$ALDH_{Tt}515$**	**1.069±0.012**	**1.016±0.011**
**$ALDH_{Tt}508$**	**1.068±0.004**	**1.001±0.003**

Supplementary Table S2. List of primers used for cloning of ALDH wildtype and mutants.

Primer Number	Description	Primer Sequence (5' – 3')
P1	Common forward primer for amplification of $ALDH_{Tt}$ from *T. thermophilus* HB27	GGAGATATACATATGCACCACCACCACCACCACCGCAAGGC GGCAGGCAAG
P2	Reverse primer for $ALDH_{Tt}$530	CTCGAATTCGGATCCTCATTAAAGCCCCAGCACC
P3	Forward inverse PCR primer for linear pET-22b+ vector	GGATCCGAATTCGAGCTC
P4	Reverse inverse PCR primer for linear pET-22b+ vector	CATATGTATATCTCCTTCTTAAAGTTAAAC
P5	Reverse primer for $ALDH_{Tt}$515	CTCGAATTCGGATCCTCATCAGTAGCCCGTGTCCAT
P6	Reverse primer for $ALDH_{Tt}$508	CTCGAATTCGGATCCTCAGAGCTGCAGCCTCCCCGAG

Supplementary Table S3: Data collection and refinement statistics

	**$ALDH_{Tt}$native**	**$ALDH_{Tt}$530 (Product-bound)**	**$ALDH_{Tt}$515 ($NADP^{+}$-bound)**	**$ALDH_{Tt}$508**
**PDB ID**	**6FJX**	**6FK3**	**6FKU**	**6FKV**
**Wavelength**	0.9686	0.9795	0.9686	0.9795
**Space group**	P 41 21 2	P 41 21 2	P 41 21 2	P 41 21 2
**Unit cell**	105.29 105.29 314.45 90 90 90	105.18 105.18 315.64 90 90 90	105.23 105.23 315.28 90 90 90	105.19 105.19 314.57 90 90 90
**Resolution range**	48.04 - 2.25 (2.33 - 2.25)	48.13 - 2.3 (2.38 - 2.30)	48.11 - 2.4 (2.48 - 2.40)	48.04 - 2.9 (3.00 - 2.90)
**R-pim**	0.09 (0.63)	0.03 (0.43)	0.05 (0.36)	0.06 (0.34)
**R-meas**	0.56 (3.24)	0.19 (2.29)	0.27 (1.90)	0.18 (0.98)
**CC1/2**	0.985 (0.75)	1 (0.88)	0.998 (0.89)	0.994 (0.79)
**Mean I/sigma(I)**	12.10 (2.21)	17.22 (2.10)	13.39 (1.86)	10.62 (1.73)
**Completeness (%)**	99.97 (99.99)	99.92 (99.90)	99.94 (99.91)	97.69 (98.86)
**Multiplicity**	30.3 (26.6)	25.0 (27.5)	26.5 (26.8)	6.3 (6.5)
**Total reflections**	2575651 (222767)	1988859 (214498)	1862508 (183700)	247104 (25364)
**Unique reflections**	84880 (8384)	79663 (7805)	70248 (6859)	39303 (3904)
**Reflections used in refinement**	84877 (8384)	79648 (7805)	70235 (6858)	39292 (3904)
**Reflections used for R-free**	2000 (198)	1999 (196)	2000 (195)	1999 (198)
**R-work**	0.189 (0.311)	0.198 (0.362)	0.183 (0.287)	0.190 (0.330)
**R-free**	0.210 (0.310)	0.243 (0.376)	0.209 (0.283)	0.229 (0.350)
**Number of non-hydrogen atoms**	8718	8759	8773	8258
**macromolecules**	8650	8632	8403	7994
**ligands**	80	99	253	77
**solvent**	292	232	180	17
**RMS(bonds)**	0.006	0.009	0.008	0.011
**RMS(angles)**	1.10	1.36	1.27	1.64
**Ramachandran favored (%)**	97.25	96.88	96.75	96.02
**Ramachandran allowed (%)**	2.75	2.94	3.25	3.98
**Ramachandran outliers (%)**	0.00	0.18	0.00	0.00
**Rotamer outliers (%)**	2.96	3.07	1.60	5.71
**Clashscore**	1.02	2.76	2.94	4.67
**Average B-factor**	45.24	57.70	44.56	57.53
**Wilson B-factor**	39.03	47.68	41.87	57.72

6FJX was obtained by merging 6 datasets.
Resolution cut-off were selected according to Karplus & Diederichs [53]; values between parentheses are for the highest resolution shell.

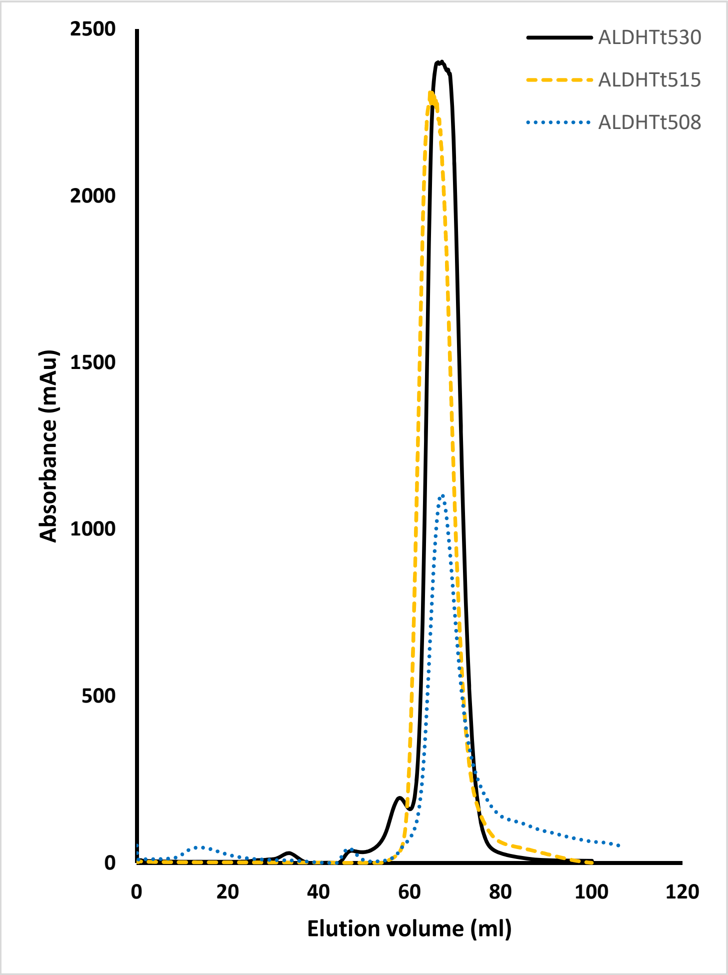

Supplementary Figure S1. Elution profiles indicate no change in oligomerization state of recombinant wildtype and truncation $ALDH_{Tt}$ mutants. The baseline for each elution was calculated as a basis to subtract the raw profile using Unicorn 5.11 software.

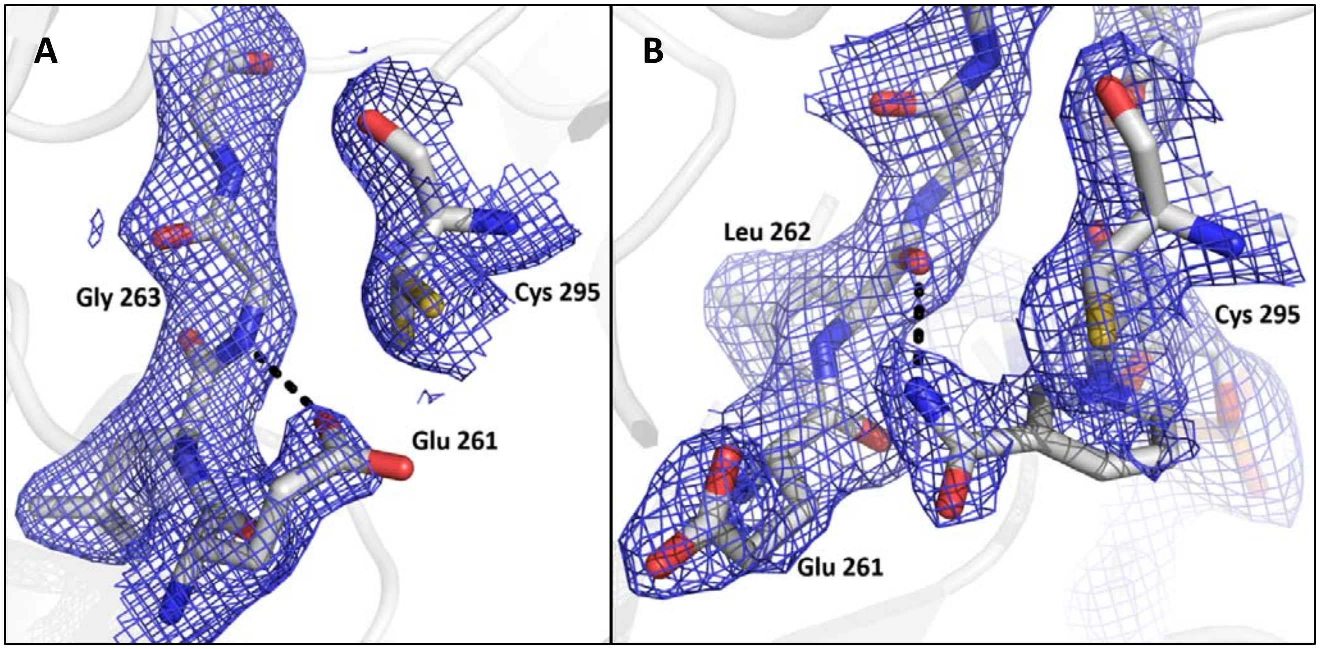

Supplementary Figure S2. Linker loop residues in apo and NADP-bound $ALDH_{Tt}$ showing different orientation of Leu262 and Gly263 amid and carbonyl group. **A:** 2FoFc map (blue mesh) contoured at 1 sigma of critical residues as observed in native and apo structures. Glu261 in the "In" position is stabilized through a hydrogen bond (dashed line) with the amid group of Gly263. **B:** 2FoFc map (blue mesh) contoured at 1 sigma of the linker loop residues as observed in NADP-bound structure. A 180 ° flip of the main chain bring the Leu262 carbonyl within hydrogen bonding distance to the carboxamide nitrogen of the cofactor. The Glu261 is push out due to cofactor binding.

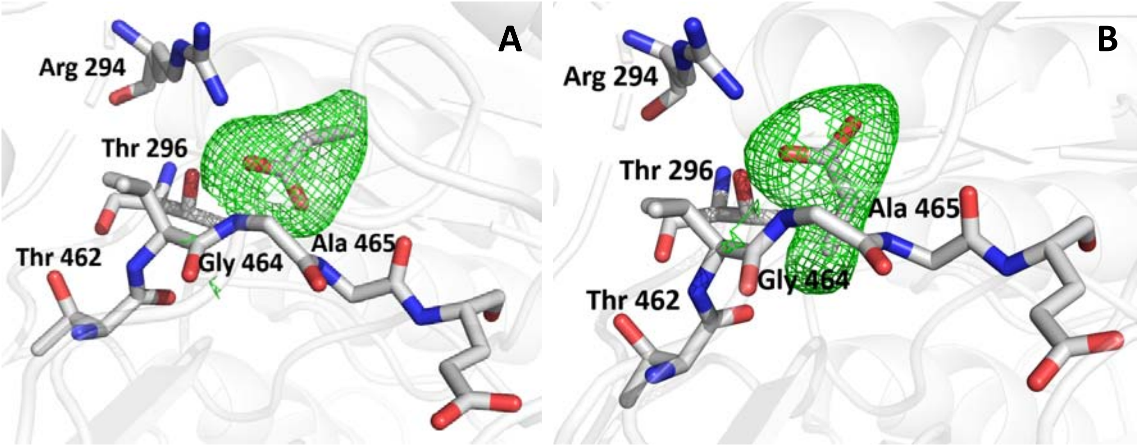

Supplementary Figure S3. Product binding in the substrate entry channel of $ALDH_{Tt}530$. The FoFc omit map (green mesh) contoured at 3 sigma of propanoic acid is shown for monomer A (**A**) and monomer B (**B**). Residues involved in propanoic acid binding are labeled and shown in sticks.

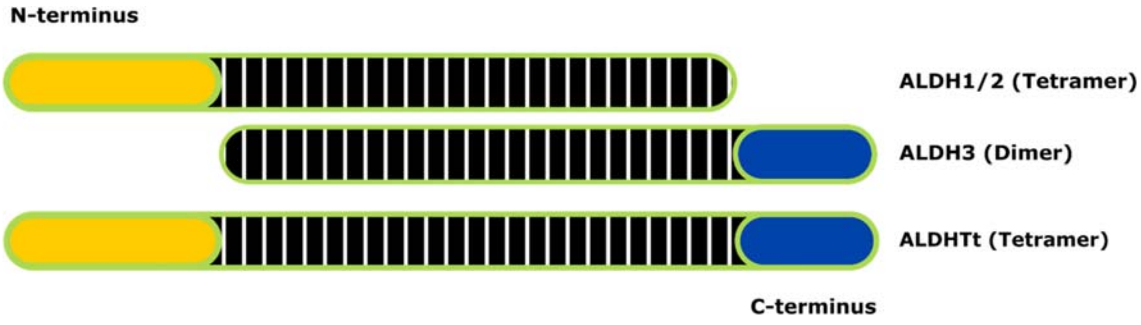

Supplementary Figure S4. Graphical representation of ALDHs architecture and terminal alignments. ALDH1/2 are defined by the presence of a full complement of N-terminal residues (yellow) and lack of C-terminal extension (Blue) whilst the opposite is the case for ALDH3. Note how $ALDH_{Tt}$ contains both defining segments allowing for a N- and C-terminal interaction between dimer pairs otherwise impossible in previously described ALDHs.

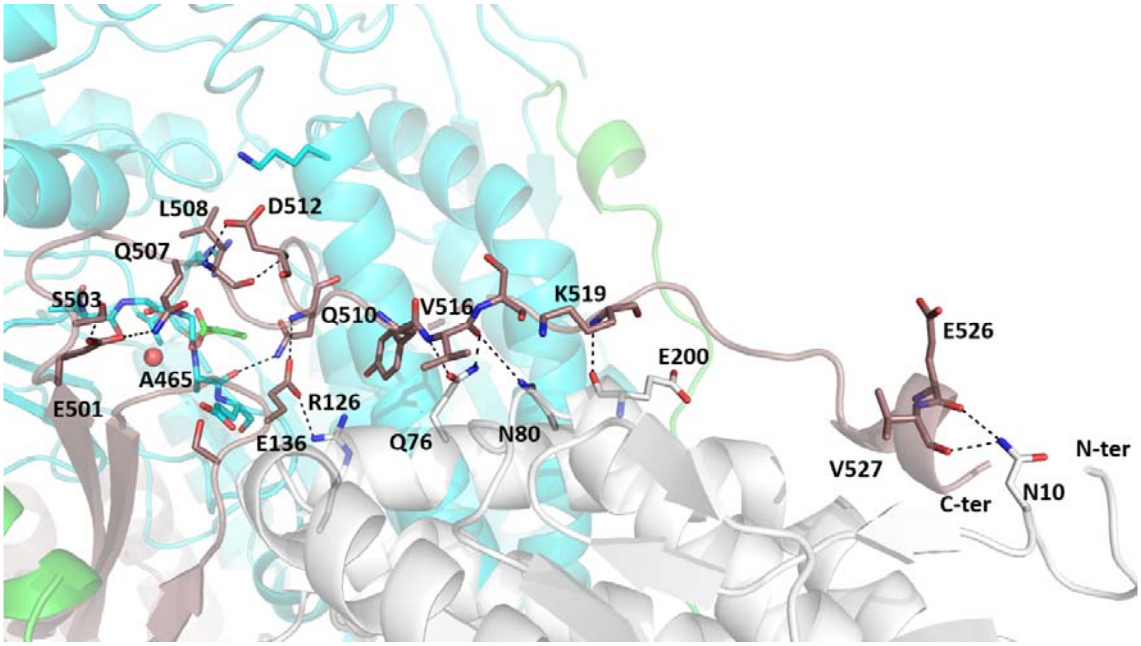

Supplementary Figure S5: A summary of the C-terminal tail interactions. All monomers are shown in cartoon and the main chain and side chain of residues involved in salt bridges and hydrogen bonds are in stick. Potential hydrogen bonds between monomers A (Brown), D (White), and B (Cyan) are shown.

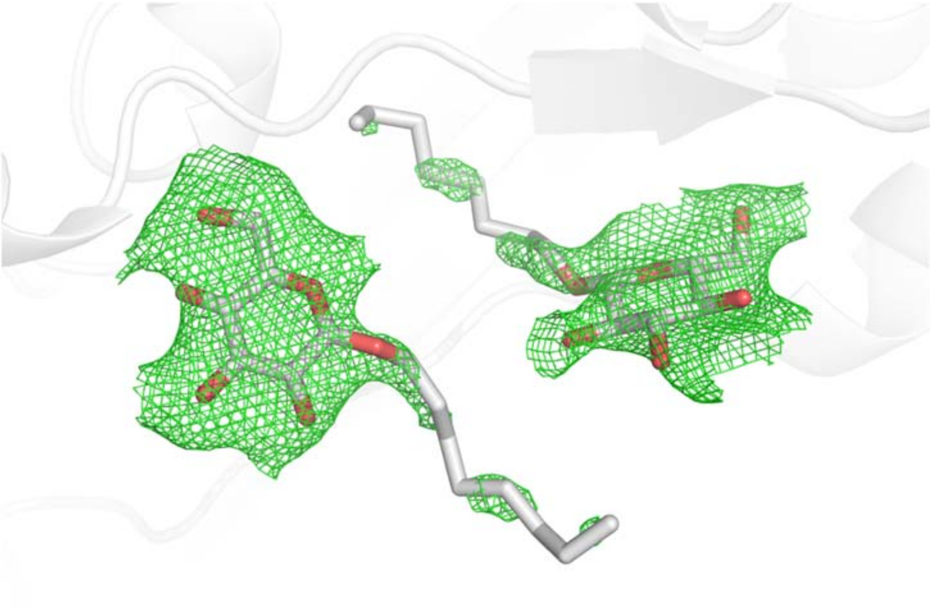

Supplementary Figure S6: FoFc omit map (green mesh) contoured at 3 sigma of bound BOG molecules in the pore of the tetrameric $ALDH_{Tt}$Native. There is a large electron density which suggest an alternative conformation for each BOG molecule in the pore.

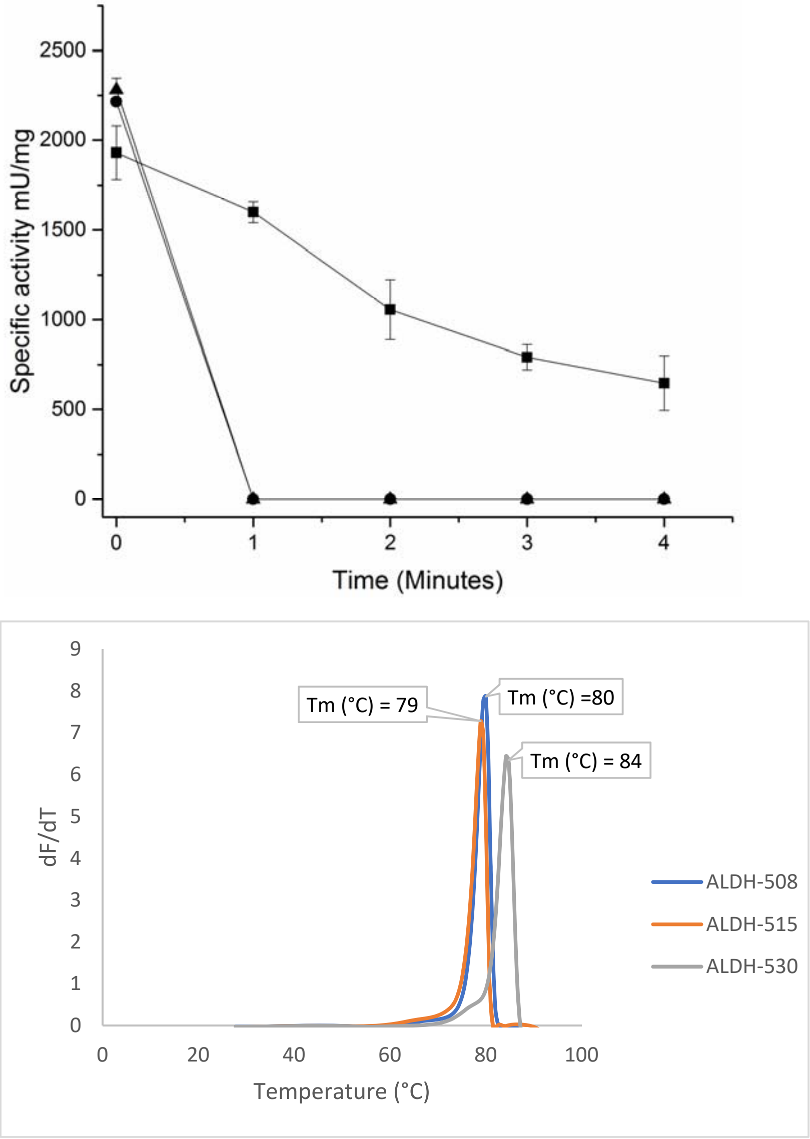

Supplementary Figure S7: Thermostability of ALDH$_{Tt}$530 measured under standard assay conditions at 85 °C. Top: specific activity of ALDH$_{Tt}$530 (■) and mutants. Note that both ALDH$_{Tt}$508 (▲) and 515 (●) rapidly lost their activity in the enzymatic assay. Bottom: Thermofluor assay indicating a lower stability of truncation mutants with T_m lower than the 85 °C.
